# Resolving the On–Off Ratio Discrepancy in Bilayer 3R-MoS_2_ FeSFETs: Dual Mechanisms of Domain Wall Engineering

**DOI:** 10.1021/acs.nanolett.5c05273

**Published:** 2026-01-29

**Authors:** Yee-Heng Teh, Horng-Tay Jeng

**Affiliations:** † Department of Physics, 34881 National Tsing Hua University, Hsinchu 30013, Taiwan; ‡ Physics Division, National Center for Theoretical Sciences, Taipei 10617, Taiwan; § Institute of Physics, Academia Sinica, Taipei 11529, Taiwan; ∥ Research Center for Semiconductor Materials and Advanced Optics, Chung Yuan Christian University, Taoyuan 32031, Taiwan

**Keywords:** Density Functional Theory (DFT) Calculations, Nonequilibrium Green Function (NEGF), Transition Metal Dichalcogenide (TMD), Ferroelectric Semiconductor Field-Effect Fransistor (FeSFET), Ferroelectric Domain Wall

## Abstract

Bilayer 3R-MoS_2_ ferroelectric semiconductor field-effect transistors (FeSFETs) commonly attributed the device’s ON-OFF switching to reversible transformation between up and down polarization, without considering the electronic properties of the domain walls (DWs) in MoS_2_. In this work, we use density functional theory combined with nonequilibrium Green’s function methods to show that DWs formation would induce electronic reconstruction at neighboring ferroelectric domains, thereby enhancing their polarization. We also found that local compressive strain in the DW can effectively increase the local conduction band minimum, drastically suppress the OFF-state current in FeSFETs. While pristine single-domain devices exhibit ON-OFF ratios of only 6.5 (armchair) and 3.8 (zigzag) at *V*_*G*_ = 0 *V*, junctions incorporating DWs can yield remarkably higher ratios of 99.1 and 33.5, respectively. This work establishes that enhanced polarization and suppressed conductance due to DWs present new avenues for improving the performance of MoS_2_-based FeSFETs.

## Graphical abstract

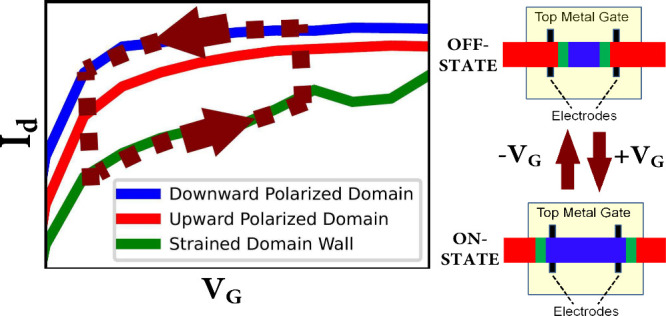

Ferroelectric semiconductor field-effect transistor (FeSFET) is a type of nonvolatile memory device where a ferroelectric material functions as the semiconductor channel.^1–3^ Two-dimensional (2D) van der Waals (vdW) ferroelectrics, such as In_2_Se_3_,^4–7^ SnS,^8^ and 3R-MoS_2_,^9–11^ have been explored for use as the semiconductor channel in FeSFETs. The atomically thin nature of these 2D-vdW ferroelectrics is advantageous for device miniaturization, while their weak out-of-plane bonding helps minimize interfacial defects during integration.

Among these materials, 3R-MoS_2_ has garnered particular attention, attributed to its ultrafast^11,12^ and high endurance^11^ switching performance, and the well-developed fabrication and transfer techniques available for MoS_2_.^13–17^ However, large discrepancy in ON-OFF current ratios^9–11^ of 3R-MoS_2_–FeSFET has been reported and the underlying reasons for this discrepancy remain unclear.

A crucial aspect for electronic applications of ferroelectrics is the behavior of their domain walls (DWs). Enhanced electrical conductivity at DWs has been reported in many 3D ferroelectrics^18–29^ and a similar phenomenon has recently been observed in 2D materials: enhanced conductivity in DWs of 2D ferroelectric SnSe,^30^ superconductivity observed in MoTe_2_ ^31^ might be attributed to its DW formation.^32^ Notably, ferroelectric DWs of LiNbO_3_,^33–41^ BiFeO_3_ ^42–45^ and Pb­(Zr,Ti)­O_3_ ^46^ have been used to construct memory units, highlighting the potential of domain walls to contribute unique electronic functionalities. Given the promise of 3R-MoS_2_ as a semiconductor channel of FeSFETs, understanding the electronic transport properties and behavior of its domain walls is of significant interest.

In this study, we investigate the influence of domain walls (DWs) on the electrical and transport properties of bilayer 3R-MoS_2_. We report a unique surface electronic reconstruction mechanism induced by the domain walls, which can dramatically enhance the polarization of neighboring ferroelectric. Moreover, strain in the DWs would induce energy shifts of the conduction band minimum (CBM), which CBM is lowered (elevated) in region of tensile (compressive) strain.

Based on calculations combining density functional theory (DFT) and nonequilibrium Green’s function (NEGF) method, we found that complete polarization switching in MoS_2_-based FeSFET yields a maximum ON/OFF current (*I*_*ON*_/*I*_*OFF*_) ratio of 6.5 (3.8) for transport along the armchair (zigzag) direction, under low drain bias of about 0.2 V. Notably, the introduction of domain wall can significantly enhance the maximum ratio to 99.1 (armchair) and 33.5 (zigzag). This comprehensive study highlights the role of domain wall in enhancing electrical polarization of bilayer 3R-MoS_2_, and boosting the *I*_*ON*_/*I*_*OFF*_ ratio of FeSFETs.

All calculations were performed using the Quantum ATK software package.^47^ Structural optimizations, formation energies, and electronic properties were investigated by using DFT. Subsequent electronic transport properties were calculated by using the NEGF method as implemented in Quantum ATK. More details about computation methods can be found in Supporting Information-1.

Our investigation begins with an analysis of the energy and polarization of bilayer MoS_2_ (arranged in a parallel orientation) across different stacking configurations. To determine the energy of each configuration, we relaxed the atomic positions along the *z*-axis while keeping the in-plane lattice constant (*a*_0_ = 3.196 Å) and the atomic positions fixed. The out-of-plane ferroelectric polarization was calculated using the berry phase method.^48^ The results [Figure 1a] indicate that the AB and BA stacking configurations are energetically favored. These configurations also exhibit the most positive (upward) and negative (downward) ferroelectric out-of-plane polarization [Figure 1b], respectively. In contrast, the saddle point (SP) and AA stacking configurations are nonpolar and less energetically stable.

**Figure 1:**
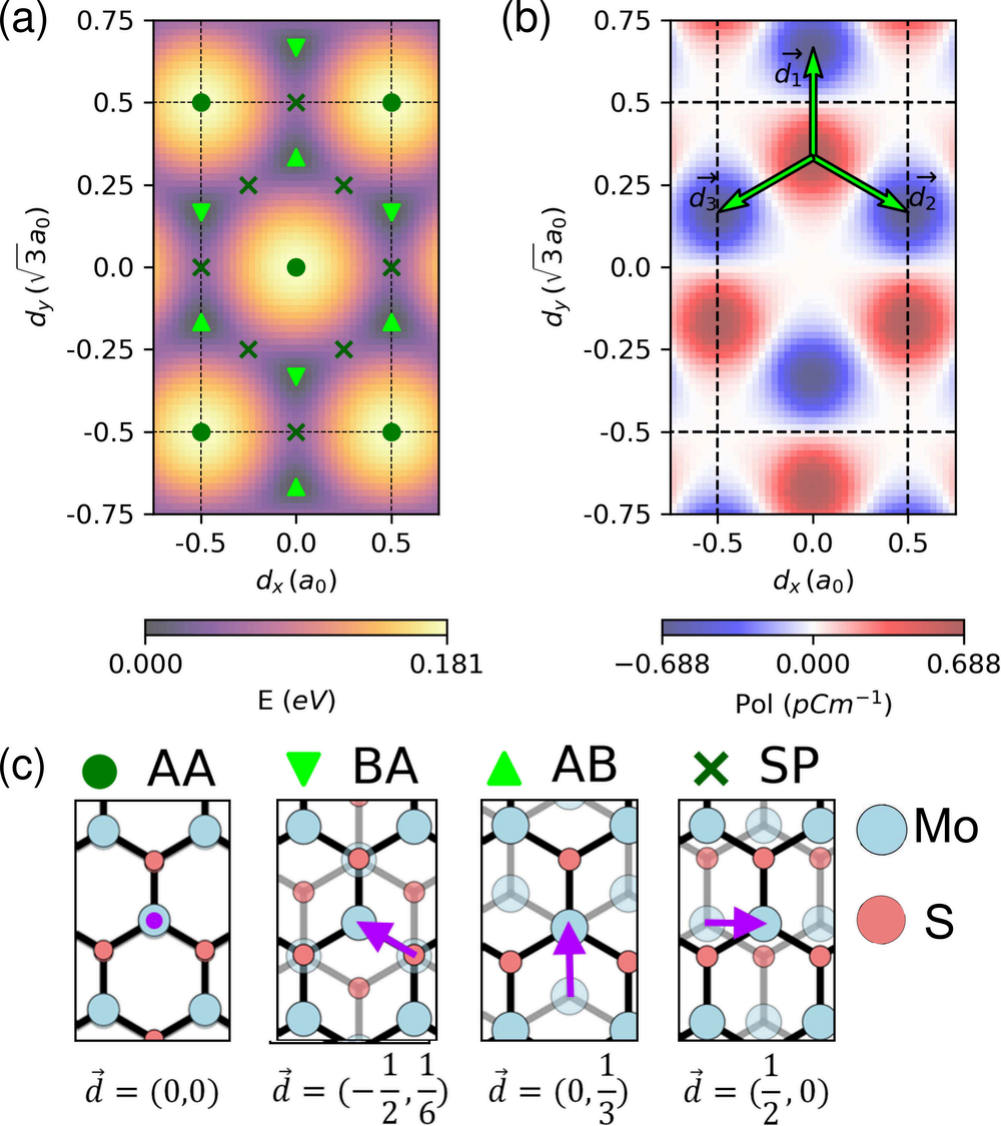
(a) Energy and (b) out of plane ferroelectric polarization as a function of displacement vector *d⃗* = (*d*_*x*_,*d*_*y*_). *d*_*x*_ and *d*_*y*_ are measured in *a*_0_ and √3*a*_0_, where *a*_0_ = 3.196 Å is the in-plane lattice constant of MoS_2_. Configurations and *d⃗* of bilayer MoS_2_ with AA, BA, AB and SP stacking order (c).

The switching of ferroelectric polarization of an entire layer from upward AB state to downward BA state requires the upper layer to slide relative to the lower layer by specific displacement vectors *d⃗* = *d⃗*_1_, *d⃗*_2_, or *d⃗*_3_. To induce formation of domain wall between opposite ferroelectric domains, we introduce incremental sliding displacement to both the lower (from 0 to $-\frac{\mathrm{d⃗}}{2}$ ) and upper (from 0 to $+\frac{\mathrm{d⃗}}{2}$ ) layers along the armchair (AC) or zigzag (ZZ) directions. The atomic positions within the domain wall region were subsequently fully relaxed to obtain the optimized structure. The domain wall energy, calculated as a function of its length [Figure S2], suggests an optimal length of approximately 10 nm, which aligns with previous experimental observations^49^ and theoretical calculations.^12,50^

Domain wall is characterized by its normal axis and the angle (θ) between the normal axis and the interlayer sliding displacement, denoted as DW_ZZ(AC),cosθ_. The domain wall energy density is found to increase in the following order [Figure S2]: ${\mathrm{DW}}_{\mathrm{AC},\pm \cos\left(0\right)}$ > ${\mathrm{DW}}_{\mathrm{ZZ},\pm \cos\left(\frac{\pi }{6}\right)}$ > ${\mathrm{DW}}_{\mathrm{AC},\pm \cos\left(\frac{\pi }{3}\right)}$ > ${\mathrm{DW}}_{\mathrm{ZZ},\cos\left(\frac{\pi }{2}\right)}$ . This trend is linked to the strain energy stored within the MoS_2_ layers: a larger magnitude of |cos θ| results in a greater strain energy. To visualize the spatial variation of atomic sliding and strain along the domain wall’s normal axis, we have plotted the top-down views of optimized domain walls and the variation of local strain within the domain wall [Figure 2a–d].

**Figure 2:**
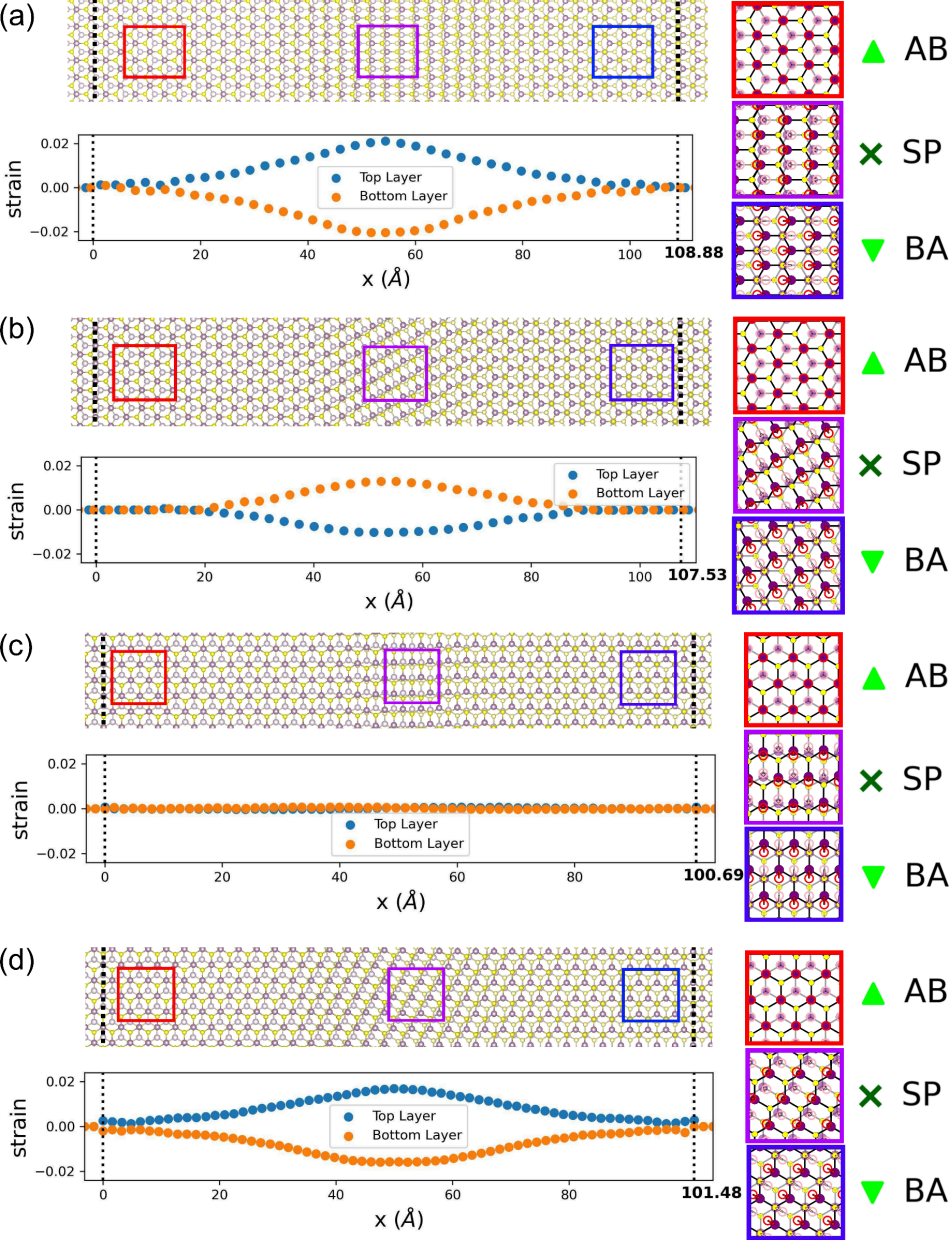
Top view illustration (top panels) of the atomic reconstruction within four different domain walls (DWs)): (a) DW_AC,cos(0)_ (b) ${\mathrm{DW}}_{\mathrm{AC},-\cos\left(\frac{\pi }{3}\right)}$ , (c) ${\mathrm{DW}}_{\mathrm{ZZ},\cos\left(\frac{\pi }{2}\right)}$ and (d) ${\mathrm{DW}}_{\mathrm{ZZ},\cos\left(\frac{\pi }{6}\right)}$ . Mo and S atoms are represented in purple and yellow circles, respectively. Selected areas are magnified with corresponding sliding displacement of the Mo atoms from their respective undistorted positions (marked with red/pink circles). The lower panel of each subfigure illustrates variation of local strain in lower and upper layer of MoS_2_, along respective domain wall’s normal axis. The method used to estimate local strain can be found in Supporting Information-2.

For the ${\mathrm{DW}}_{\mathrm{ZZ},\pm \cos\left(\frac{\pi }{2}\right)}$ configuration, the atomic sliding is perpendicular to the domain wall’s normal axis, leading to negligible strain (less than 0.1%) within the domain wall. On the other hand, ${\mathrm{DW}}_{\mathrm{AC},\pm \cos\left(\frac{\pi }{3}\right)}$ , ${\mathrm{DW}}_{\mathrm{ZZ},\pm \cos\left(\frac{\pi }{6}\right)}$ and DW_AC,±cos(0)_ have progressively higher maximum compressive (tensile) strain: 1.0 (1.3)% < 1.6 (1.7)% < 2.0 (2.1)%. It is noteworthy that both the atomic sliding and strain are more pronounced near the center of the domain wall, rather than being uniformly distributed. This can be understood as balance between the deformation energy, which is minimized by spreading the deformation, and the stacking energy, which is minimize by localizing the transition.^51^

Having defined the domain wall structure for various angle θ, we now investigate the polarization profile along the normal axis of the domain wall. Considering the out-of-plane spontaneous polarization of sliding ferroelectric arise from an interlayer charge transfer mechanism,^52–54^ we calculate the areal density of ferroelectric dipole moment as *P* = −∫*zδρ*(*z*) d*z*,^55,56^ where *δρ* is charge density transfer between the layers of a single bilayer MoS_2_, measured in electron number density. The out-of-plane ferroelectric polarizations with different stacking configurations were recalculated using the charge transfer method [Figure S3] and contrast the results from the berry phase method [Figure 1b]. These two methods show good agreement, although the charge transfer method consistently yields a ferroelectric polarization that is about 10% lower.

We then construct periodic supercells comprising alternating ferroelectric domains connected by domain walls (DWs) with different sliding displacements, as illustrated in [Figure 2a–d]. Dipole moment densities (*P*) across upward ferroelectric domain of these supercells are shown in Figure 3a–d [full polarization profile across the supercells can be found in Supporting Information-4]. A key observation is that while the atomic positions within the ferroelectric domains are identical with those in their pristine (single-domain) counterparts, the magnitude of *P* within these domains is significantly larger.

**Figure 3:**
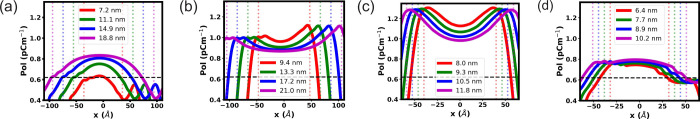
Spatial profiles of the dipole moment density across upward-polarized ferroelectric domains calculated using the charge transfer method. Each panel shows a domain connected by a different type of domain wall (DW): (a) DW_AC,±cos(0)_, (b) ${\mathrm{DW}}_{\mathrm{AC},\pm \cos\left(\frac{\pi }{3}\right)}$ , (c) ${\mathrm{DW}}_{\mathrm{ZZ},\pm \cos\left(\frac{\pi }{2}\right)}$ , and (d) ${\mathrm{DW}}_{\mathrm{ZZ},\pm \cos\left(\frac{\pi }{6}\right)}$ . The solid colored lines show the calculated polarization within the ferroelectric domains, which are the regions bound by the vertical dotted lines. The dashed black line indicates the reference polarization value of a pristine domain.

The enhancement and spatial distribution of *P* in ferroelectric domains is found to be correlated with the angle θ and width of domain walls. For the supercell with DW_AC,±cos(0)_[Figure 3a], the magnitude of *P* is weaker near the domain wall and reaches its maximum value at the center of the domain. This central maximum also increases with domain width, converging to the extrapolated value of 0.91 pC m^–1^ [Figure S5a]. Conversely, a supercell with ${\mathrm{DW}}_{\mathrm{ZZ},\cos\left(\frac{\pi }{2}\right)}$ ­[Figure 3c] has its magnitude of *P* peaks at the domain wall boundary and decreases to a local minimum at the center of the domain. This central minimum value decreases as the domain width increases, extrapolating to a near pristine polarization value of 0.68 pC m^–1^ at infinite width [Figure S5b]. For domain wall configurations ${\mathrm{DW}}_{\mathrm{AC},\pm \cos\left(\frac{\pi }{3}\right)}$ and ${\mathrm{DW}}_{\mathrm{ZZ},\pm \cos\left(\frac{\pi }{6}\right)}$ , the atomic sliding displacement is neither perfectly parallel nor perpendicular to the domain wall normal. Consequently, their polarization profiles appear as a superposition of the profiles observed for ${\mathrm{DW}}_{\mathrm{ZZ},\cos\left(\frac{\pi }{2}\right)}$ and DW_AC,±cos(0)_. Enhancement of *P* can be attributed to surface charge reconstruction, which details of mechanism is provided in Supporting Information-4.

Figure 4a,b shows the conduction band minimum (CBM) and valence band maximum (VBM) of Molybdenum (Mo) atoms in the lower layer of bilayer MoS_2_ supercells, resolved along the domain wall’s normal axis. The CBM/VBM of the upper layer atoms can be inferred from symmetry considerations: the CBM/VBM of Mo atoms in the upper layer of a upward ferroelectric domain (or of DW_AC/*ZZ*,±cos(θ)_ region) is equivalent to that in the lower layer of the downward ferroelectric domain (or of DW_AC/*ZZ*,∓cos(θ)_ region), and vice versa. Our analysis focuses on the CBM for several key reasons: it is primarily derived from Mo orbitals; [Figure S8], its density of states is significantly larger than that of the VBM [Figure S8], and its energy level is highly sensitive to the local atomic configuration. The latter two points strongly suggest that n-doped MoS_2_ would be a superior channel material for high-performance ferroelectric semiconductor field-effect transistors (FeSFETs), promising a larger ON-OFF ratio.

**Figure 4:**
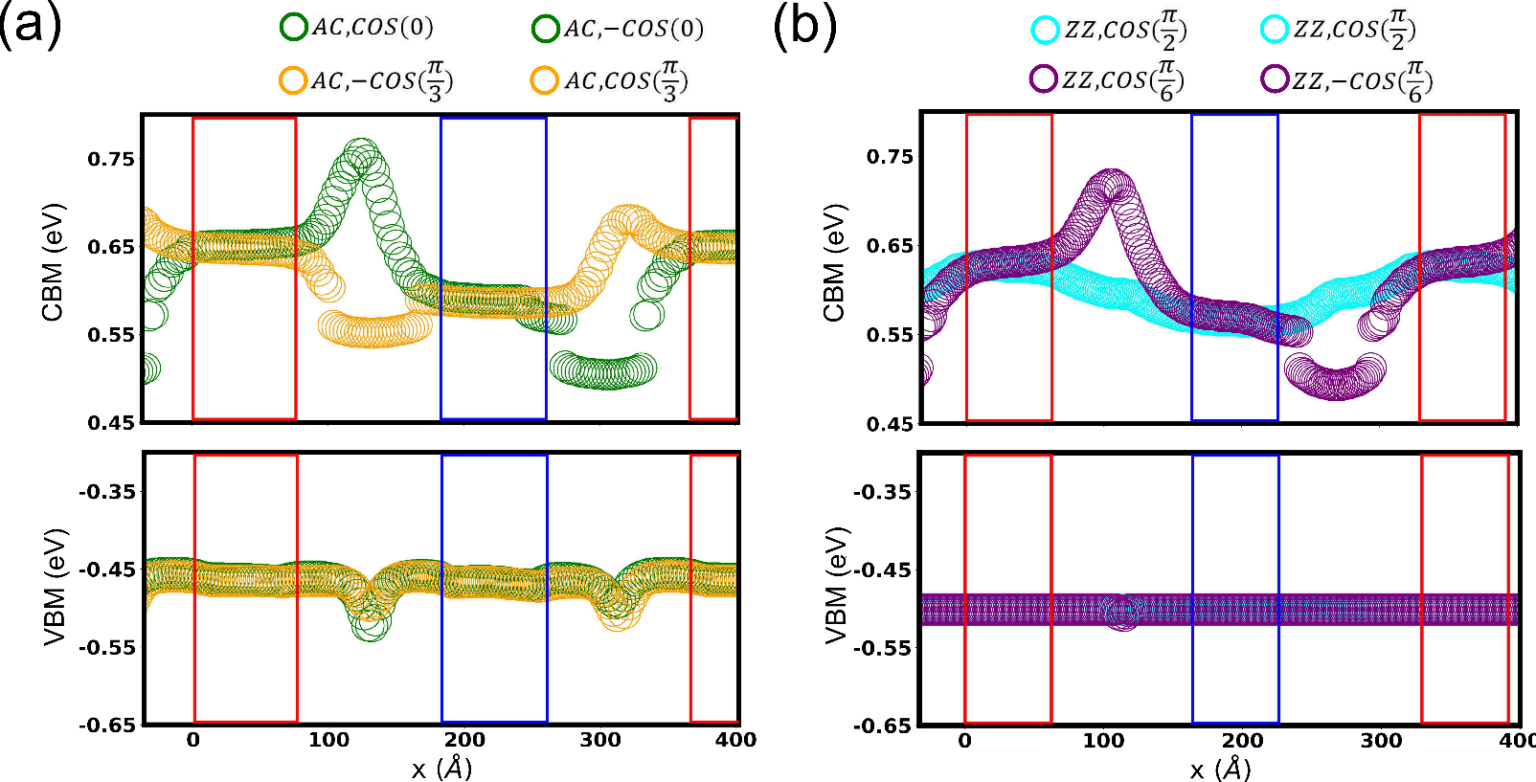
Conduction band minimum (CBM) and valence band maximum (VBM) of molybdenum (Mo) atoms in the lower layer of bilayer MoS_2_ supercells, resolved along the domain wall’s normal axis. Panels (a) and (b) correspond to supercells with a normal axis directed along armchair and zigzag direction, respectively. The CBM varies significantly within the domain walls compared with the polarized domains, while the VBM remains relatively stable across the entire supercell.

Across all configurations, the CBM difference between the upward and downward ferroelectric domains is consistently found to be approximately 70 meV, matching the value in the pristine material. However, the electronic structure within the domain walls is highly dependent on their type. For the ${\mathrm{DW}}_{\mathrm{ZZ},\cos\left(\frac{\pi }{2}\right)}$ , the CBM assumes an intermediate energy between the two domains. In contrast, for the DW_AC,±cos(0)_, ${\mathrm{DW}}_{\mathrm{AC},\pm \cos\left(\frac{\pi }{3}\right)}$ and ${\mathrm{DW}}_{\mathrm{ZZ},\cos\left(\pm \frac{\pi }{6}\right)}$ , the CBM extrema are located at the saddle points within the walls themselves. The CBM is systematically lowered in regions of tensile strain and elevated in regions of compressive strain. This strain-induced modulation of the CBM results in energy differences that scale with the magnitude of the local strain, following the trend ${\mathrm{DW}}_{\mathrm{ZZ},\cos\left(\frac{\pi }{2}\right)}\left(70\;\mathrm{meV}\right)$ < ${\mathrm{DW}}_{\mathrm{AC},\pm \cos\left(\frac{\pi }{3}\right)}\left(150\;\mathrm{meV}\right)$ < ${\mathrm{DW}}_{\mathrm{ZZ},\pm \cos\left(\frac{\pi }{6}\right)}\left(250\;\mathrm{meV}\right)$ < ${\mathrm{DW}}_{\mathrm{AC},\pm \cos\left(0\right)}\left(260\;\mathrm{meV}\right)$ .

Figure 5a illustrates the schematic diagram of device architectures used for our transport calculations. The electrodes, highlighted by the white box, consist of five atomic layers of lithium. This material was chosen due to its small lattice mismatch (less than 2%) with the MoS_2_ channel. A 5.0 nm long high-*k*/metal gate (HKMG), featuring a 1.0 nm thick dielectric (*k* = 5.2), is placed over the central scattering region. This gate modulates the Fermi level within the junction:^57^ a positive (negative) gate voltage raises (lowers) the Fermi level, effectively creating n-type (p-type) doping.

**Figure 5:**
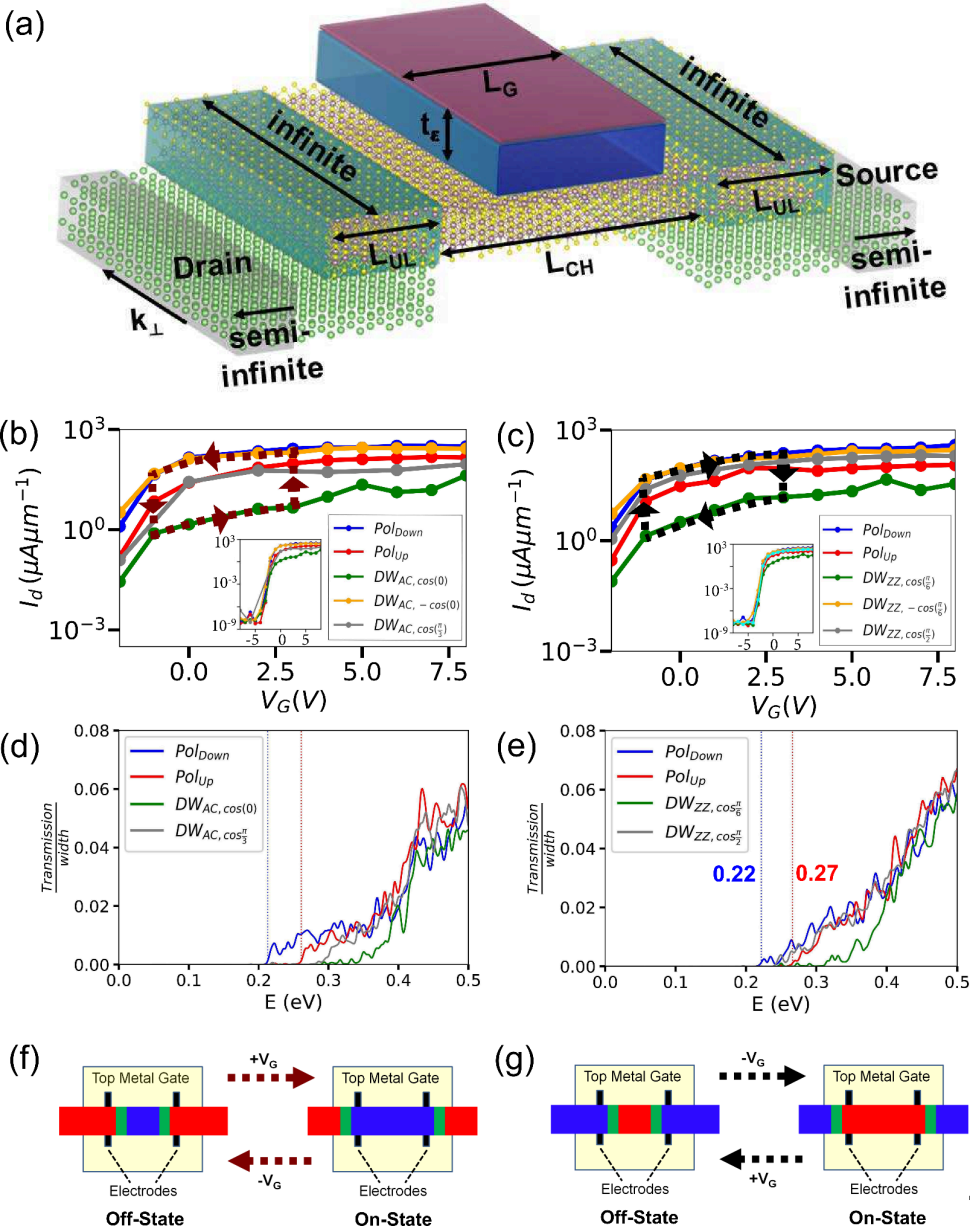
(a) A schematic of the gated bilayer MoS_2_ FeSFET is shown. The regions where the Li electrodes overlap with the MoS_2_ consist of bilayer 3R-MoS_2_ with pristine configuration, while the central channel region is varied in different calculations to include different domain walls. Following parameters were used in NEGF calculations: Length of metal gate (*L*_*G*_) = 5.0 nm, thickness of dielectric (*t*_ϵ_) = 1.0 nm, channel’s length (*L*_*CH*_) = 11.0 nm, length of overlapped region between MoS_2_ and electrode (*L*_*UL*_) = 2.8 nm (armchair) *or* 2.6 nm (zigzag). Transfer curves along (b) armchair and (c) zigzag axes across various domain wall junctions and single domains (up/down polarization) are plotted and magnified at −1 V ≤ *V*_*G*_ ≤ 8 V, while the full transfer curves (−8 V ≤ *V*_*G*_ ≤ 8 V) are shown in the inset figures. An anticlockwise and a clockwise hysteresis loops are shown in panel (b) and (c) respectively. Transmission functions at *V*_*G*_ = 0 V along (d) armchair and (e) zigzag axes across various junctions are plotted. The edge of transmission function of fully downward (upward)-polarized junction is shown in vertical blue­(red) dotted line. (f,g) Schematic of ON-OFF switching via propagation of domain wall into or out of the junction, results in anticlockwise (panel (b)) or clockwise (panel (c)) hysteresis loop.

We calculated the transfer curves [Figure 5b,c] across various domain wall junctions, including single domains (up/down polarization along armchair and zigzag) and several domain walls: DW_AC,±cos(0)_, ${\mathrm{DW}}_{\mathrm{AC},\pm \cos\left(\frac{\pi }{3}\right)}$ , ${\mathrm{DW}}_{\mathrm{ZZ},\cos\left(\frac{\pi }{2}\right)}$ and ${\mathrm{DW}}_{\mathrm{ZZ},\pm \cos\left(\frac{\pi }{6}\right)}$ . Electrode voltages (*V*_*L*_ = 0.0 V) and drain bias (*V*_*d*_ = 0.2 V, 0.21 V along armchair and zigzag axis, respectively) were chosen to maximize the *I*_*ON*_/*I*_*OFF*_, while ensuring that *I*_*ON*_ exceeded a reference value of 40 μA/μm (an experimental *I*_*ON*_ of a short-channel (25 nm) monolayer MoS_2_–FET under low drain bias of 0.05 V^58^). As detailed in Supporting Information-6, a larger bias voltage could yield a higher *I*_*ON*_ but concurrently reduce the *I*_*ON*_/*I*_*OFF*_ ratio.

The drain current across the junction was calculated for a gate voltage (*V*_*G*_) range of −8 to 8 V with electrons being dominant charge carriers. For negative gate voltages (−8 V ≤ *V*_*G*_ < 0 V), the Fermi level is shifted down toward the valence band, depleting the channel of free carriers and causing a sharp reduction in drain current (*I*_*d*_). Conversely, applying a positive gate voltage (0 V < *V*_*G*_ ≤ 8 V) results in only a modest increase in current. This behavior is attributed to the partial gate geometry: the overall transmission becomes limited by the series resistance of the ungated sections of the channel, which are not modulated by the HKMG. The following discussion of transfer curves would focus on transmission in range of *V*_*G*_ ≥ −1 V as due to their significant larger *I*_*d*_.

The current in the purely downward-polarized domain (*I*_*down*_) is consistently larger than current through the purely upward-polarized domain (*I*_*up*_), for transport either along the armchair or zigzag axis. The behavior of the current across different domain wall configurations is as follows: (a) *I*_*d*_ across domain walls with a compressed lower layer (DW_ZZ/AC,cosθ>0_) are lower in magnitude than *I*_*down*_ and *I*_*up*_. (b) *I*_*d*_ across the ${\mathrm{DW}}_{\mathrm{ZZ},\cos\left(\frac{\pi }{2}\right)}$ configuration shows an intermediate value, falling between *I*_*up*_ and *I*_*down*_. These findings indicate that channel configurations with a higher CBM in their lower MoS_2_ layer exhibit a lower conductivity. This is because the lower layer serves as the primary channel for current transmission, and a higher CBM corresponds to a higher edge of the transmission function, thus impeding the current flow [Supporting Information-7]. Interestingly, while domain walls with an expanded lower layer (DW_ZZ/AC,cosθ<0_) have a lower CBM than the downward-polarized domain, the *I*_*d*_ across them is almost equal to *I*_*down*_ for *V*_*G*_ ≥ 0 V. This occurs because these domain walls form at the interface between downward- and upward-polarized domains, and the overall conductivity becomes bottlenecked by the lower conductivity of the upward- polarized domain.

Previous studies^1,59^ of FeSFETs using an In_2_Se_3_ channel have demonstrated impressive performance. These devices exhibit a distinct shift in their threshold voltage (*V*_*th*_) and large ON/OFF ratio (*I*_*ON*_/*I*_*OFF*_ ≈ 10^8^), which is attributed to the significant change in surface bound charge during polarization switching. Replicating this performance in FeSFETs based on bilayer MoS_2_ is challenging. This difficulty arises from the significantly weaker intrinsic ferroelectric polarization of bilayer MoS_2_, which is approximately 30 times smaller than that of monolayer In_2_Se_3_.^60^

The consequence of this weak polarization is evident in the device’s transmission function: upon complete polarization switching, the edge of the transmission shifts by only about 50 meV [Figure 5d,e]. This energy shift is comparable to the thermal energy at room temperature, which is insufficient to effectively modulate the channel from a high-resistance state to a low-resistance state [Supporting Information-7]. As a result, the bilayer MoS_2_ FeSFET exhibits a very low intrinsic *I*_*ON*_/*I*_*OFF*_ (defined here as *I*_*down*_/*I*_*up*_) across a wide range of gate voltages (−1 V ≤ *V*_*G*_ ≤ 8 V). Specifically, the ratio falls between 2.1 and 3.8 for transport along the zigzag axis and between 2.1 and 6.5 for transport along the armchair axis. These values are orders of magnitude lower than the *I*_*ON*_/*I*_*OFF*_ typically achieved in conventional, atomically thin MoS_2_ FETs (which can exceed 10^7^ ^58^). However, they are consistent with previously reported values for bilayer MoS_2_ FeSFETs, which also show very modest *I*_*ON*_/*I*_*OFF*_ (around 1.1).^9,11^

The ability to manipulate domain walls with a gate voltage offers a promising route to enhancing FeSFET performance. A gate-induced vertical electric field (*E*_⊥_) can drive the propagation of sliding domain walls to expand ferroelectric domains aligned with the field.^12,50^ Building on this principle, we propose the *V*_*G*_ can be used to control the number of domain walls within the device channel to engineer the OFF-state conductivity [Figure 5f,g]. Since domain walls with compressive strain at their lower layers can act as high-resistance scattering regions, intentionally increasing their number can create a highly resistive OFF-state, leading to a much larger *I*_*ON*_/*I*_*OFF*_. We note that a larger range of gate voltage would likely enlarge the memory window since the strain of domain walls are proportional to domain walls’ kinetic friction (minimally strained ${\mathrm{DW}}_{\mathrm{ZZ},\cos\left(\frac{\pi }{2}\right)}$ on the other hand exhibits nearly zero damping motion);^12,61^ thus, a larger range of gate voltage is required to propagate them into or out of the channel.

This hypothesis is supported by our calculations, which show that incorporating even a single domain wall can significantly increase the *I*_*ON*_/*I*_*OFF*_ ratio. Specifically, the junction containing DW_AC,cos(0)_ records a maximum *I*_*ON*_/*I*_*OFF*_ ratio of 99.1 at *V*_*G*_ = 0 V, which slightly decreases to 88.9 at *V*_*G*_ = −1 V. On the other hand, the junction containing ${\mathrm{DW}}_{\mathrm{ZZ},\cos\left(\frac{\pi }{6}\right)}$ records a maximum *I*_*ON*_/*I*_*OFF*_ ratio of 33.5 at *V*_*G*_ = −1 V, which slightly decreases to 27.9 at *V*_*G*_ = 0 V. This multiwall mechanism also provides a plausible explanation for recent experimental results: a long-channel (μm-scale) bilayer MoS_2_ FeSFET characterized with compressed and strained domain walls was reported to achieve an *I*_*ON*_/*I*_*OFF*_ greater than 10^5^,^10^ a value significantly higher than what our models predict for a single domain wall. We suggest that this impressive performance is likely due to the presence of multiple compressed domain walls within the long channel [Supporting Information-8] and higher resistance domain walls formed between AA and AB­(BA) stacked MoS_2_ [Supporting Information-9], which collectively suppress the OFF-state current far more effectively. Conversely, FeSFETs based on exfoliated bilayer 3R-MoS_2_ channels reported with low *I*_*ON*_/*I*_*OFF*_ (around 1.1),^9,11^ are likely dominated by the energetically most stable domain wall, ${\mathrm{DW}}_{\mathrm{ZZ},\cos\left(\frac{\pi }{2}\right)}$ .^62,63^ Our results indicate that this particular domain wall is actually more conductive than the upward-polarized “OFF” state, rendering it a less effective OFF-state (maximum $\frac{I_{\mathrm{ON}}}{I_{\mathrm{OFF}}}$ < 2).

In conclusion, we have employed DFT and NEGF methods to investigate the electronic and transport properties of ferroelectric bilayer MoS_2_, containing four distinct types of domain walls (DWs). Our results show that the formation of DWs induces a surface charge reconstruction that significantly enhances the ferroelectric polarization (*P*) in the neighboring domains. The enhancement of *P* is dependent on both the width of the ferroelectric domain and the type of connected DWs. Specifically, ferroelectric domain connected by ${\mathrm{DW}}_{\mathrm{ZZ},\cos\left(\frac{\pi }{2}\right)}$ shows larger enhancement of *P* at smaller width of ferroelectric domain; in contrast, ferroelectric domain connected by DW_AC,cos(0)_ shows stronger enhancement of *P* at larger width.

The formation of DWs also directly impacts the device’s transport characteristics. DWs that induce compressive strain in their lower layer drastically suppress the FeSFET’s conductance. This suppression is most pronounced at a gate voltage range of −1 V ≤ *V*_*G*_ ≤ 0 V, where the maximum current ratio (*I*_*down*_/*I*_DW_) for devices with DW_AC,cos(0)_ and ${\mathrm{DW}}_{\mathrm{ZZ},\cos\left(\frac{\pi }{6}\right)}$ are 99.1 and 33.5, far exceeding the intrinsic (*I*_*down*_/*I*_*up*_) ratios of 6.5 and 3.8, for transportation along armchair and zigzag axis, respectively. We note that the presence of multiple domain walls along the transport axis can lead to even more substantial *I*_*OFF*_ suppression, further increasing the *I*_*ON*_/*I*_*OFF*_.

Our findings also provide a compelling explanation for the substantial discrepancy in experimentally measured *I*_*ON*_/*I*_*OFF*_. Exfoliated MoS_2_–FeSFETs,^9,11^ expected to feature predominantly low-energy ${\mathrm{DW}}_{\mathrm{ZZ},\cos\left(\frac{\pi }{2}\right)}$ that exhibit conductivity similar to that of pristine ferroelectric domains, consistently yielding low *I*_*ON*_/*I*_*OFF*_ (around 1.1). In contrast, the naturally strained MoS_2_–FeSFETs fabricated via CVD methods^10^ are more prone to forming highly resistive strained domain walls, which accounts for their significantly enhanced *I*_*ON*_/*I*_*OFF*_. This work underscores domain wall engineering as a potent strategy for boosting MoS_2_-based FeSFET performance, pushing capabilities beyond the material’s intrinsic limits.

## Supplementary Material



## Notes

Computational methods, domain wall energy density, polarization density calculated using charge transfer method, enhancement of polarization due to atomic and electronic reconstruction, band structures of pristine bilayer 3R-MoS_2_, *I*_*ON*_ and *I*_*ON*_/*I*_*OFF*_ as a function of gate voltage and drain bias, transmission profile across FeSFET, Electrical current across multidomain wall junction, electrical current across domain wall formed between AB- and AA-stacked MoS_2_ (PDF)
