# Supplementary material for: Resolving the On–Off Ratio Discrepancy in Bilayer 3R-MoS2 FeSFETs: Dual Mechanisms of Domain Wall Engineering
Source: Nano Lett. 2026 Jan 29;26(5):1673–81. doi: 10.1021/acs.nanolett.5c05273 (PMC12904094; doi:10.1021/acs.nanolett.5c05273)
Supplement: Supplementary file 1 [file nl5c05273_si_001.pdf]

Supporting Information

Resolving the On-Off Ratio Discrepancy in Bilayer 3R-MoS_2 FeSFETs: Dual Mechanisms of Domain Wall Engineering

Yee-Heng Teh[1] and Horng-Tay Jeng[1, 2, 3, 4, *]

[1] *Department of Physics, National Tsing Hua University, Hsinchu 30013, Taiwan*
[2] *Physics Division, National Center for Theoretical Sciences, Taipei 10617, Taiwan*
[3] *Institute of Physics, Academia Sinica, Taipei 11529, Taiwan*
[4] *Research Center for Semiconductor Materials and Advanced Optics, Chung Yuan Christian University, Taoyuan 32031, Taiwan*

1. COMPUTATIONAL METHODS

All calculations were performed using the Quantum ATK software package [1]. Structural optimizations, formation energies, and electronic properties were investigated using density functional theory (DFT). The generalized gradient approximation (GGA) with the Perdew-Burke-Ernzerhof (PBE) functional was employed for the exchange-correlation energy [2]. The valence electron wavefunctions were described using Dojo pseudopotential and linear combination of atomic orbitals (LCAO) basis set.

Density mesh and reciprocal cutoff energy were set to 2200 eV and 35000 eV, respectively. Monkhorst-Pack k-point mesh of $10 \times 10 \times 1$ were used for pristine bilayer MoS_2 calculations, and the k-point mesh was rescaled to maintain a comparable grid density in the Brillouin Zone (BZ) for supercell calculation. Additionally, long-range van der Waals interactions were incorporated using Grimme's D2 semi-empirical dispersion correction [3]. To avoid the interactions of adjacent slabs, Dirichlet and Neumann boundary conditions are used at lower and upper boundary along the z-axisa , with vacuum spacing of 20 Å to eliminate any spurious interactions between periodic images. It is important to note that Dirichlet and Neumann boundary conditions enable us to obtain fully converged polarization value that is insensitive to the vacuum thickness [Fig S1].

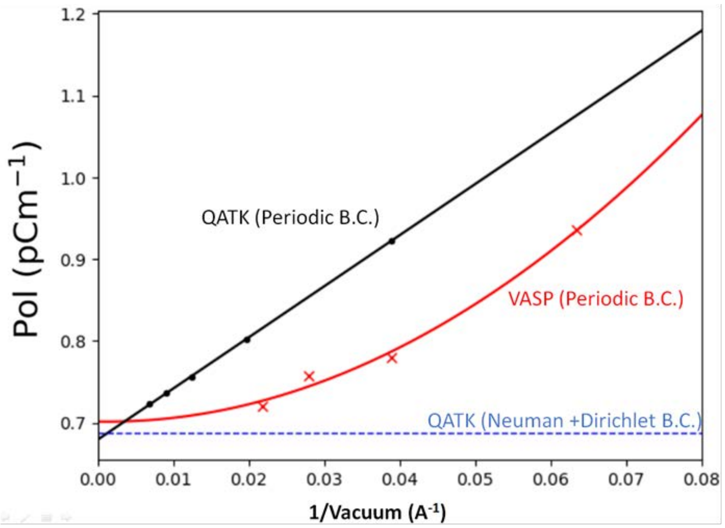

Fig. S1. Polarization of a bilayer MoS_2 calculated using three distinct computational setups: (a) VASP with periodic boundary conditions (PBC) along the z-axis. (b) QuantumATKwith periodic boundary conditions (PBC) along the z-axis. (c) QuantumATKwith Dirichlet and Neumann boundary conditions along the z-axis .

* jeng@phys.nthu.edu.tw

The atomic structures of pristine bilayer MoS_2 and 5-layers (111) surface of body-centered cubic Lithium metal were relaxed until the residual force on each atom was below 0.02 eV/ Å, and the total energy was converged to within 10^{-5} eV. For supercells consist of opposite polarized ferroelectric domains connected by domain walls (DWs), atoms within the ferroelectric domains with length of 11(7) unit cells along the armchair (zigzag) axis were fixed in positions, while the positions of atoms within the domain wall were fully relaxed.

Subsequent electronic transport properties were calculated using the non-equilibrium Green's function (NEGF) method as implemented in Quantum ATK. The drain current (I) was calculated using the Landauer-Büttiker formula[4]:

$$\begin{aligned} I(V_L, V_R, T) = & \frac{e}{h} \int_{k_\perp, E} T_\sigma(k_\perp, E, V_R - V_L) \\ & [f(\frac{E - (E_F - V_L)}{k_B T}) - \\ & f(\frac{E - (E_F - V_R)}{k_B T})] dE \end{aligned} \tag{1}$$

where f is the Fermi-Dirac distribution, E_F is fermi energy of the device, $V_{L(R)}$ is the left (right) electrode voltage, k_B is Boltzmann constant, T is the temperature of the electrodes and T_σ is the transmission coefficient. The calculation employs the zero-bias approximation, where the transmission function, $T_\sigma(E)$, is computed at equilibrium and assumed to be independent of the bias voltage. We solve Eq.1 under varying gate voltage, which gate voltage is modeled as a fixed potential in solving the Poisson equation, not a physical electrode.

2. DOMAIN WALL ENERGY DENSITY

Domain wall energy density (U_{DW}) as function of length of domain wall (l_{DW}) is calculated as:

$$U_{DW}(l_{DW}) = (E_{SC} - N_{MoS_2}E_{UC})/(w * t) \tag{2}$$

where E_{SC} is the energy of supercell consists of ferroelectric domains connected by domain wall, E_{UC} is energy of a unit cell ferroelectric MoS_2, N_{MoS_2} is the number of MoS_2 in supercell, w is width of supercell and t is the thickness of bilayer MoS_2 (12.2Å).

The domain wall energy density is found to increase in the following order[Fig S2]: $DW_{AC,\pm cos(0)} > DW_{ZZ,\pm cos(\frac{\pi}{6})} > DW_{AC,\pm cos(\frac{\pi}{3})} > DW_{ZZ,cos(\frac{\pi}{2})}$. This trend is linked to the strain energy stored within the MoS_2 layers: a larger magnitude of $|cos\theta|$ results in greater strain energy.

Local strain (ϵ) arises from atomic reconstruction due to domain wall's formation are shown in Fig 2 in main text. ϵ is calculated as:

$$\epsilon(\frac{x_i + x_j}{2}) = |(x_i - x_j)|/(l_0) \tag{3}$$

where $x_{i,j}$ are positions of two nearest Mo atoms (i, j) projected along the domain wall's normal axis and l_0 is 2.768 Å (1.598 Å), which is the distance between two nearest Mo atoms in an unstrained lattice, projected along the armchair (zigzag) axis.

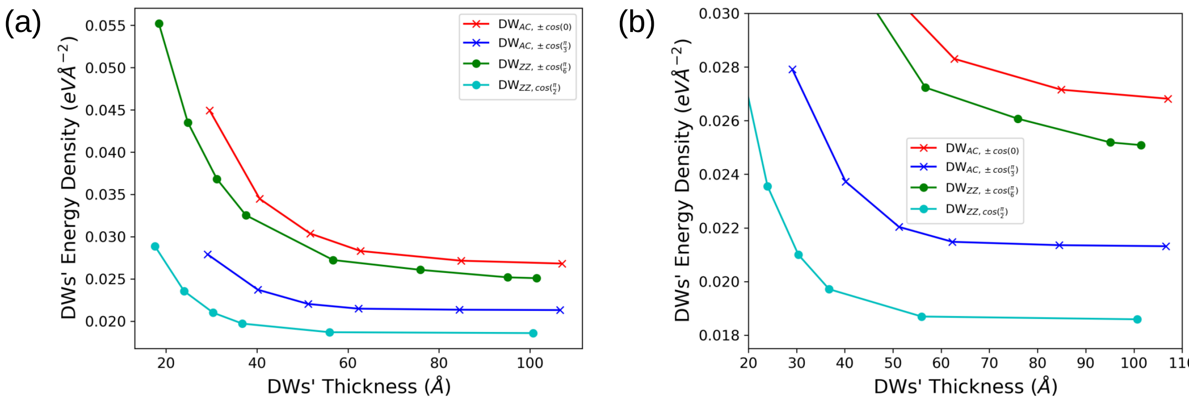

Fig. S2. (a) Domain wall (DW) energy density of $DW_{AC,\pm cos(0)}$, $DW_{AC,\pm cos(\frac{\pi}{3})}$, $DW_{ZZ,cos(\frac{\pi}{2})}$ and $DW_{ZZ,\pm cos(\frac{\pi}{6})}$. (b) Figure (a) is amplified near the converged energy.

3. POLARIZATION DENSITY CALCULATED USING CHARGE TRANSFER METHOD

The out-of-plane ferroelectric polarization with different stacking configurations were recalculated using the charge transfer method [Fig S3a]. To understand how the charge density transfer, $\delta\rho$ give rise to ferroelectric polarization, the side view of the $\delta\rho$ in bilayer MoS_2 with AB stacking (upward-polarized) is plotted in [Fig S3b]. A significant variation in $\delta\rho$ is observed at the interface between the layers, indicating a transfer of negative charge from the upper to the lower layer, which give rise to upward polarization according to the expression $P = \int z\delta\rho(z)dz$. The discontinuity of spontaneous polarization at the upper and lower surface would create positive and negative surface bound charge, respectively [5]. Such surface bound charge is however difficult to be visualized in [Fig S3b] since they are much smaller in magnitude compared to electron density transfer from surface S atoms to their neighboring Mo atoms. To illustrate the mentioned surface bound charge, the difference in $\delta\rho$ between the AB (upward-polarized) and BA (downward-polarized) stacking configurations of MoS_2 is also plotted [Fig S3c]. The sign of this difference ($\delta\rho_{AB} - \delta\rho_{BA}$) alternates along the out-of-plane axis, showing the top surface of the upward-polarized AB MoS_2 exhibits a more positive bound charge than its bottom surface.

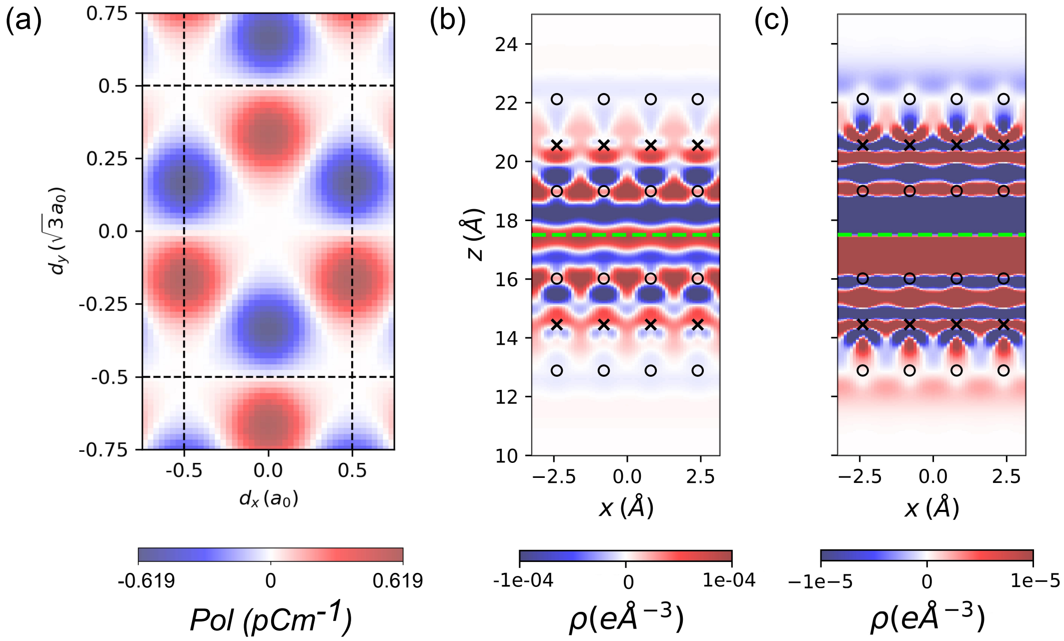

Fig. S3. (a) Out of plane ferroelectric polarization calculated using charge transfer method, as function of displacement vector $\overrightarrow{d} = (d_x, d_y)$. (b) Side view of the $\delta\rho$ in bilayer MoS_2 with AB stacking (upward-polarized). (c) Side view of difference in $\delta\rho$ between the AB and BA stacking configurations of MoS_2 ($\delta\rho_{AB} - \delta\rho_{BA}$).

4. ENHANCEMENT OF POLARIZATION DUE TO ATOMIC AND ELECTRONIC RECONSTRUCTION

We construct periodic supercells comprising alternating ferroelectric domains, connected by domain walls (DWs) with different sliding displacements, as illustrated in Fig 2 of the main text. Each supercell contains two domains of opposite polarization, oriented along the zig-zag (or armchair) axis, and separated by two distinct DWs, each approximately 10 nm wide. The first type of DW ($DW_{ZZ(AC),\pm cos\theta}$), illustrated in Fig 2 of the main text, connects a upward to a downward polarized domain along the positive x-axis. The second type ($DW_{ZZ(AC),\mp cos\theta},$), which connects the upward to the downward polarized domain along the negative x-axis, is an inverse of the first along the z-axis. Consequently, the upper and lower layers of this $DW_{ZZ(AC),\pm cos\theta}$ are equivalent to the lower and upper layers of $DW_{ZZ(AC),\mp cos\theta}$, respectively.

The dipole moment density, P of these supercells are plotted along the domain wall's normal axis [Fig S4a-d]. Fig S4a-d illustrates the calculated dipole moment density (P) across the supercells. The regions enclosed by red and blue rectangles denote the upward and downward ferroelectric domains, respectively. Two methods were used to determine P: a charge transfer method (solid black lines) and an estimation based on the relative displacement of Molybdenum (Mo) atoms between the upper and lower MoS_2 layers (green crosses) [Fig S2a]. A key observation is that while the atomic positions within the ferroelectric domains are identical to those in their pristine (single-domain) counterparts, the magnitude of P within these domains is significantly larger.

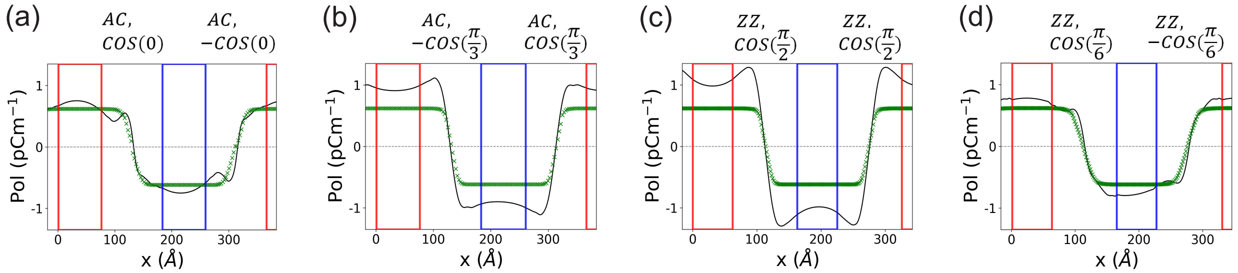

Fig. S4. Dipole moment density of periodic supercells with alternating ferroelectric domains, where upward and downward ferroelectric domains are enclosed in red and blue rectangles, respectively. The domains are connected by four different domain walls (DWs): (a) $DW_{AC,\pm cos(0)}$ (b) $DW_{AC,\mp cos(\frac{\pi}{3})}$, (c) $DW_{ZZ,\pm cos(\frac{\pi}{2})}$ and (d) $DW_{ZZ,\pm cos(\frac{\pi}{6})}$. The dipole moment density is plotted as a solid black line (calculated via the charge transfer method) and as green crosses (estimated from the sliding displacements of Molybdenum atoms).

The enhancement and spatial distribution of P is found to be correlated with the angle θ. For supercells with $DW_{ZZ,cos(\frac{\pi}{2})}$, the polarization profile [Fig S4c] is antisymmetric with respect to the center of the domain wall. The magnitude of P peaks at the domain wall-ferroelectric domain boundary and diminishes to a local minimum at the center of the ferroelectric domain. This local minimum value decreases as the domain width increases [Fig 3c], extrapolating to near pristine polarization value of 0.68 pCm^{-1} at infinite width [Fig S5a].

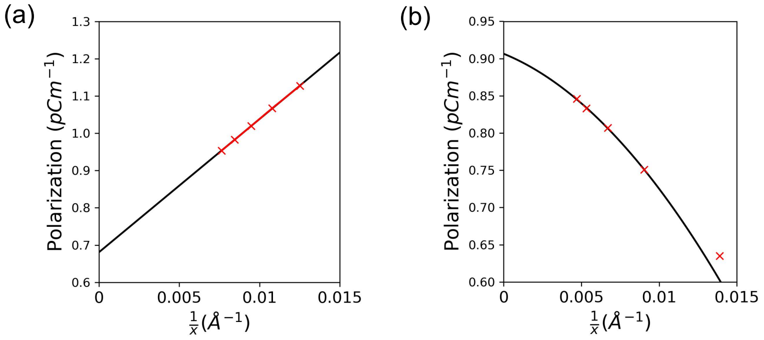

Fig. S5. (a) Central minimum and (b) central maximum dipole moment density of ferroelectric domain as function of domain wall's width. The ferroelectric domains are connected by $DW_{ZZ,\pm cos(\frac{\pi}{2})}$ and $DW_{AC,\pm cos(0)}$, respectively.

In contrast, for supercells containing $DW_{AC,\pm cos(0)}$, the polarization distribution [Fig S4a] is asymmetric. A local

minimum (or maximum) in P is observed near the boundary between $DW_{AC,cos(0)}$ (or $DW_{AC,-cos(0)}$) and the adjacent upward (or downward) ferroelectric domain, while the polarization reaches its maximum value at the center of the domains. This central maximum also increases with domain width, converging to the extrapolated value of 0.91 pCm^{-1} [Fig S5b].

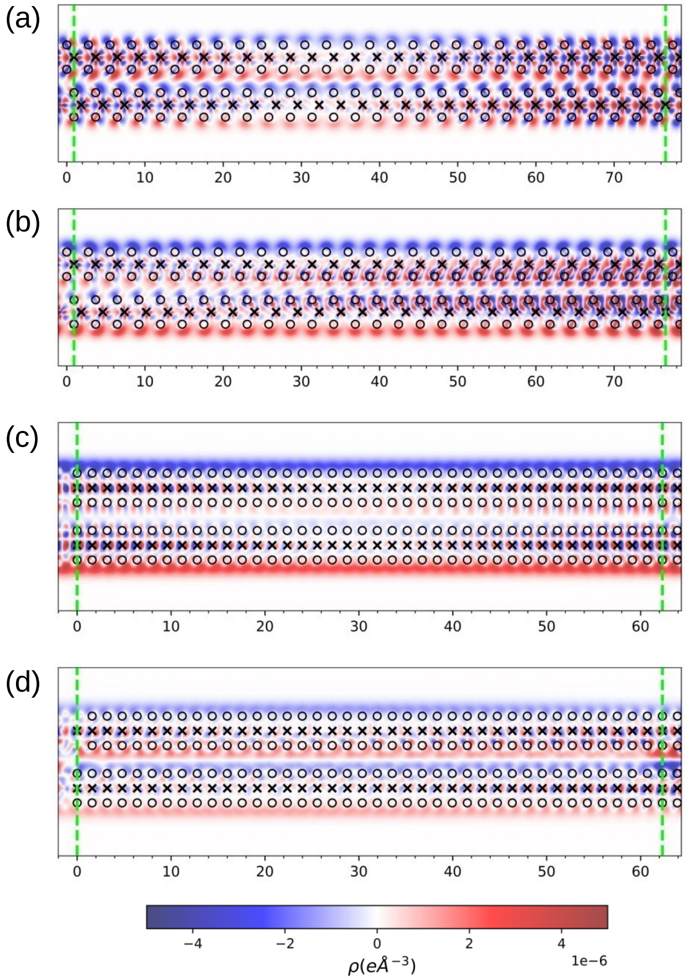

Fig. S6. Differential charge density transfer ($\Delta(\delta\rho)$) is defined as the difference between the upward ferroelectric domain in supercell and its pristine counterpart ($\delta\rho_{AB(supercell)}\delta\rho_{AB(pristine)}$). $\Delta(\delta\rho)$ is plotted along the domain walls' normal axis, for supercells connected with four different domain walls (DWs): (a) $DW_{AC,\pm cos(0)}$ (b) $DW_{AC,\mp cos(\frac{\pi}{3})}$, (c) $DW_{ZZ,\pm cos(\frac{\pi}{2})}$ and (d) $DW_{ZZ,\pm cos(\frac{\pi}{6})}$.

For the remaining domain wall configurations, $DW_{AC,\pm cos(\frac{\pi}{3})}$ and $DW_{ZZ,\pm cos(\frac{\pi}{6})}$, the atomic sliding displacement is neither perfectly parallel nor perpendicular to the domain wall normal. Consequently, their polarization profiles appear as a superposition of the profiles observed for $DW_{ZZ,cos(\frac{\pi}{2})}$ and $DW_{AC,\pm cos(0)}$.

The enhancement of P is attributed to electronic reconstruction at the domain interfaces and surfaces. To quantify this effect, the differential charge density transfer ($\Delta(\delta\rho)$), defined as the difference between the upward ferroelectric domain in supercell and its pristine counterpart ($\delta\rho_{AB(supercell)} - \delta\rho_{AB}$), is plotted in Fig S6a-d.

The most pronounced electronic reconstruction, on the order of 10^{-6} eÅ^{-3}, is observed at the MoS_2 interlayer interface and at the top and bottom surfaces of the bilayer slab. At the interlayer interface, reconstruction induces a transfer of electrons from the lower to upper MoS_2 layer. This charge transfer opposes the primary dipole moment, thereby reducing the magnitude of P. However, this reduction is negligible, as the initial charge transfer at the interface is approximately 10^{-4} eÅ^{-3} [Fig S3b], which is two orders of magnitude larger than the perturbation from the reconstruction. Conversely, the surface reconstruction effects amplify the net polarization. At the surfaces, electronic reconstruction causes a depletion of electron density at top surface and an accumulation at the bottom surface. This contribution is significant because the difference of $\delta\rho$ between the two surfaces in the pristine cell

($\sim 10^{-6}$ eÅ^{-3}) is comparable in magnitude to the reconstruction-induced charge redistribution.

The physical origin of the observed surface electronic reconstruction is the differential response of the material's top and bottom surfaces to the reconstruction within the domain wall. This phenomenon manifests differently depending on the angles (θ) between the sliding displacement and the domain wall's normal axis. In the supercell contains $DW_{ZZ,cos(\frac{\pi}{2})}$, electronic reconstruction at the top surface generates an in-plane dipole moment centered at the saddle point [Fig S7a]. This dipole, directed toward the upward ferroelectric domain, is formed by an accumulation of electrons at the boundary with the downward-polarized domain and a depletion at the boundary with the upward-polarized domain. This charge redistribution directly enhances the out-of-plane polarization of the adjacent domains. This conclusion is substantiated by the strong correlation between the average surface electron transfer profile and the overall polarization profile [Fig S4c and Fig S7a, respectively].

In contrast, the atomic and electronic reconstruction in the $DW_{AC,\pm cos(0)}$ domain wall differs significantly from that in the $DW_{ZZ,cos(\frac{\pi}{2})}$ type. The saddle point region in the upper layer of $DW_{AC,cos(0)}$ is subjected to large tensile strain, which results in a depletion of surface electrons in this area. Consequently, electrons are transferred away from the saddle point, increasing the transferred electron density on the top surface of the adjacent downward ferroelectric domain [FigS7c] and thereby enhancing its out-of-plane polarization. Conversely, the electron depletion at the saddle point creates a local maximum in transferred electron density at the boundary with the upward ferroelectric domain, resulting in a local minimum in out-of-plane polarization. An analogous charge transfer mechanism is observed in the upper layer of the $DW_{AC,-cos(0)}$ structure, which is equivalent to the lower layer of $DW_{AC,cos(0)}$. In this case, compressive strain at the saddle point leads to an accumulation of surface electrons. This, in turn, depletes transferred electrons in the neighboring upward ferroelectric domain (enhancing its polarization) and establishes a local minimum in transferred electron density at the boundary with the downward ferroelectric domain, which produces a local polarization maximum [FigS4a].

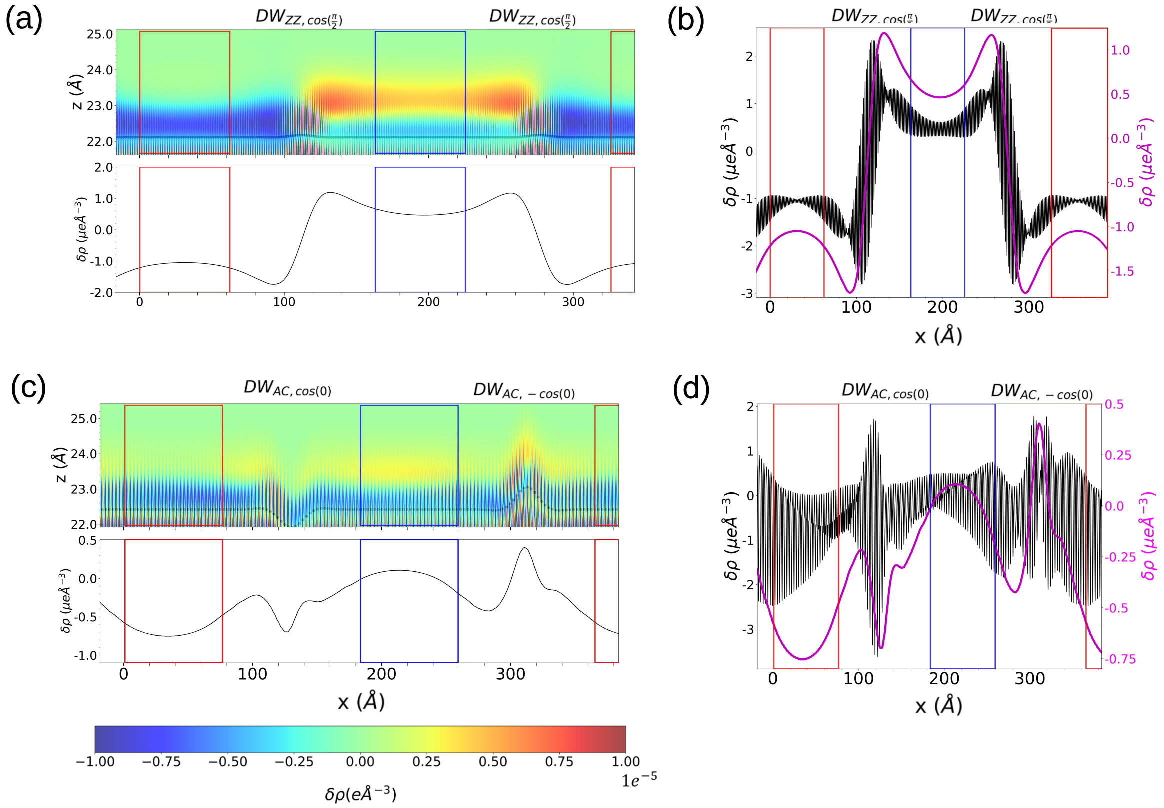

Fig. S7. Upper panels of (a) and (c) visualize the charge density transfer ($\delta\rho$) near the surface of supercells containing the domain walls (DWs) $DW_{ZZ,\pm cos(\frac{\pi}{2})}$ and $DW_{AC,\pm cos(0)}$, respectively. In these figures, upward and downward ferroelectric domains are enclosed in red and blue rectangles, and gray circles indicate the positions of surface sulfur atoms. Lower panels (a) and (c) plot the average $\delta\rho$ (convolved with a Gaussian Kernel) for the corresponding supercells, calculated within the region extending from surface sulfur atoms to 5 Å above the surface. The comparison of $\delta\rho$ with (magenta lines) and without (black lines) Gaussian convolution were shown at panels (b) and (d).

5. BAND STRUCTURES OF PRISTINE BILAYER 3R-MOS$_2$

Band gap of band structures increases (decreases) with compressive (tensile) strain. Compressive(tensile) strain would elevate(lower) the energy level of CBM along GX, and lower(elevate) energy level of CBM along SY.

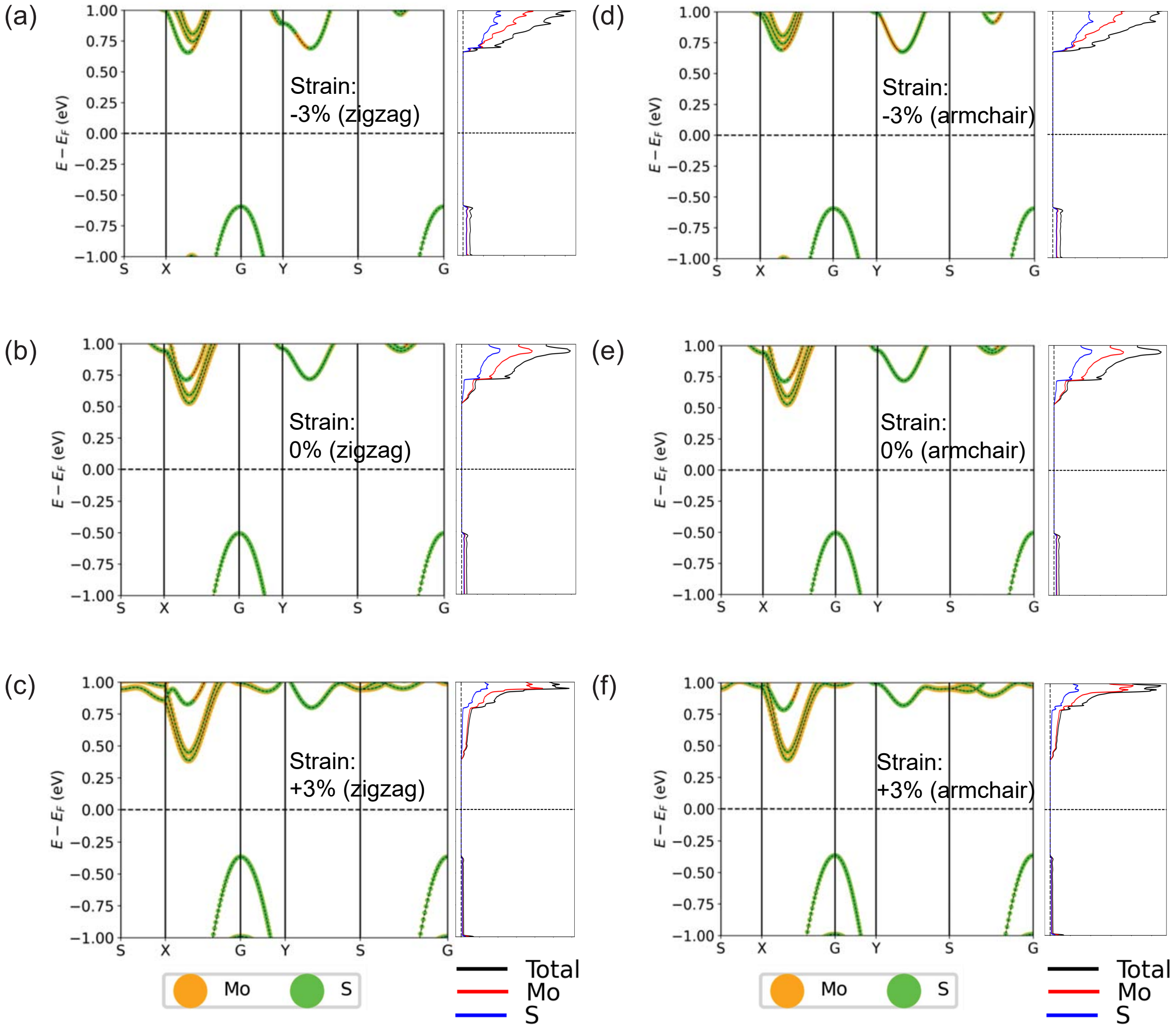

Fig. S8. Panels (a), (b) and (c) present orbital decomposed band structures of bilayer 3R-MoS$_2$ subjected to strain of −3.0%, 0.0% and+3.0%, applied along the zigzag axis. Corresponding band structures with strain applied along armchair axis are presented in panels (d), (e) and (f). Projected density of states at different strain conditions were plotted alongside corresponding panels.

6. I_{ON} AND I_{ON}/I_{OFF} AS FUNCTION OF GATE VOLTAGE AND DRAIN BIAS.

By setting $V_{Left} = 0\,V$, we calculate the ratio of current across downward-polarized domain (I_{down}) to upward polarized domain and several high resistivity domain walls, as functions of gate voltage (V_G) and bias voltage (V_d) [Fig S9a-d]. At $-3\,V \leq V_G \leq 8\,V$, larger V_d results in larger I_{down} but significant smaller ratio. We also observe greater suppression of $I_{DW_{AC,cos(0)}}$ and $I_{DW_{ZZ,cos(\frac{\pi}{6})}}$ at negative $V_G \leq -4\,V$, leading to much larger ratio of $\frac{I_{down}}{I_{DW_{AC,cos(0)}}}$ and $\frac{I_{down}}{I_{DW_{ZZ,cos(\frac{\pi}{6})}}}$ (near order of 10^3) at large drain bias. At $V_G = 0\,V$, significant amount of electrons are transmitted across the upper layers of the high resistivity domain walls [Fig S10f], as opposed to I_{down} which mainly transmitted through the lower layer [Fig S10d]. At negative V_G, the gate field is stronger at upper layer and module their doping more effectively, thus suppressing $I_{DW_{AC,cos(0)}}$ and $I_{DW_{ZZ,cos(\frac{\pi}{6})}}$ more effectively.

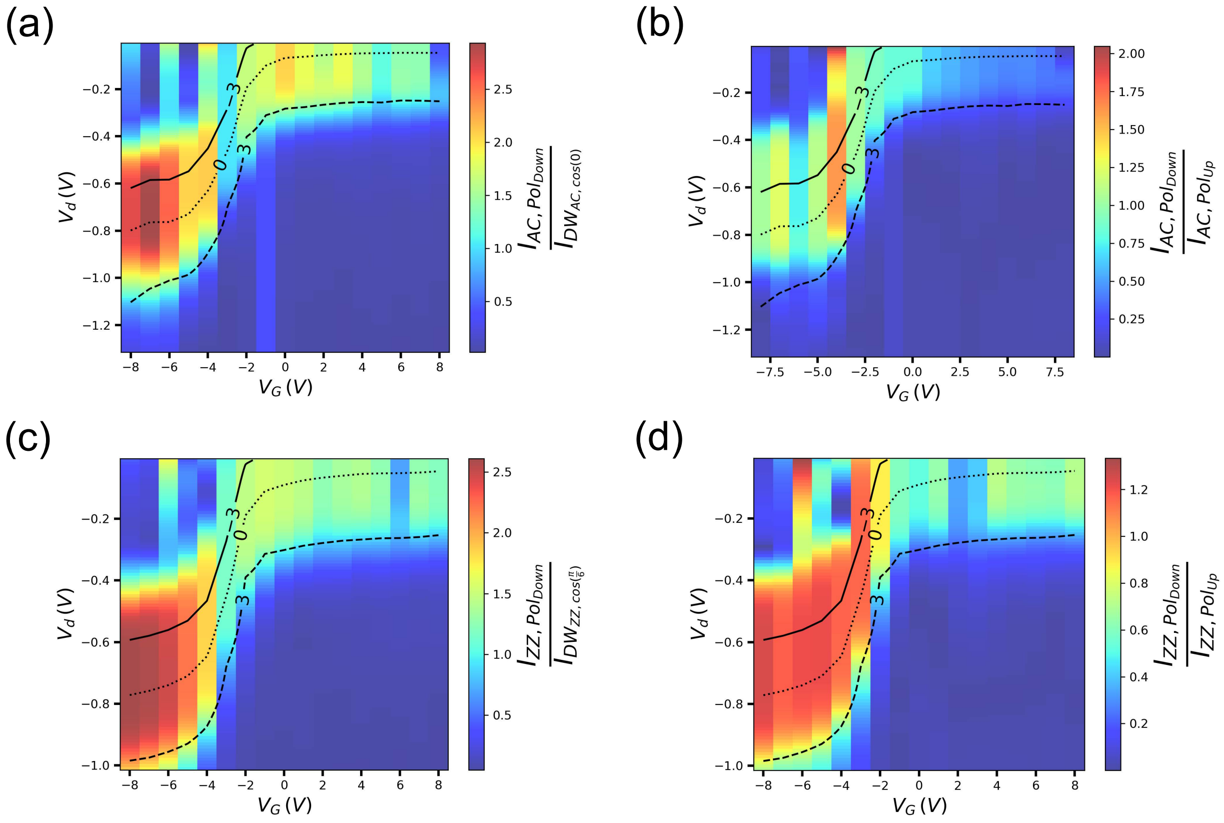

Fig. S9. Ratio of current across downward-polarized domain (I_{down}) to different junctions' configuration: (a) $DW_{AC,cos(0)}$, (b) upward-polarized domain with transportation along armchair axis; (c) $DW_{ZZ,cos(\frac{\pi}{6})}$, (d) upward-polarized domain with transportation along zigzag axis. The values of ratio are presented in heatmap and the I_{down} is plotted in contour lines.

7. TRANSMISSION PROFILE ACROSS FESFET

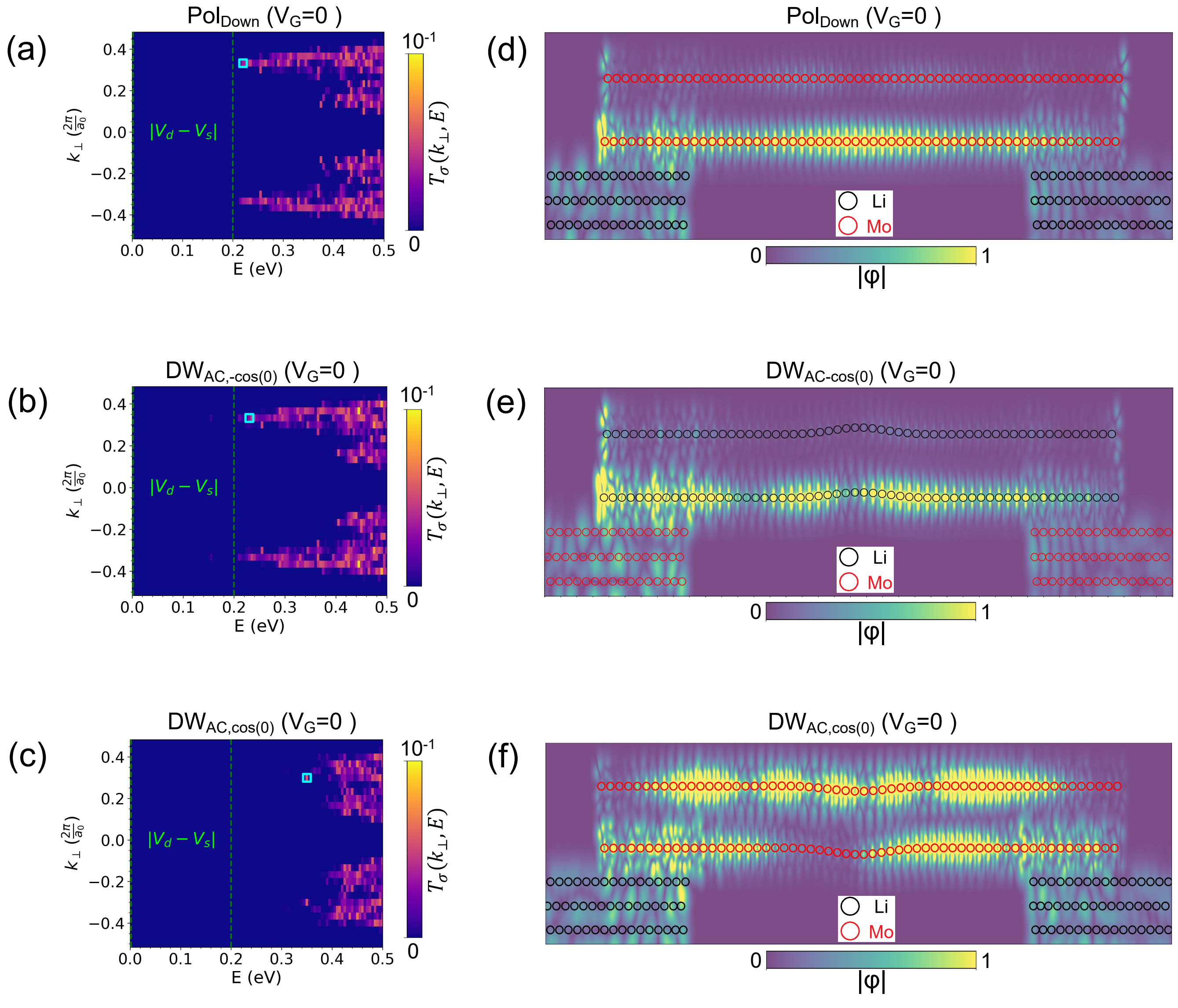

Fig. S10. k-space resolved transmission coefficient of junction consists of bilayer MoS_2 with (a) single down-polarized domain, (b) $DW_{AC,-cos(0)}$ and (c) $DW_{AC,cos(0)}$ at $V_G = 0V$. The amplitude of transmission eigenstate at the eigenchannel of (E, k) point labeled on panel (a-c) are shown at panel (d-f), respectively.

The k-space resolved transmission spectrum, $T_\sigma(k_\perp, E)$, provides the physical origin for the observed conductance trends[Fig 5b-c in main text]: downward-polarized domain is the most conductive, junctions with tensile strain at lower layer ($DW_{AC,-cos(0)}$ and $DW_{ZZ,-cos(\frac{\pi}{6})}$ exhibit intermediate current (between I_{up} and I_{down}), junctions with compressed lower layer ($DW_{AC,cos(0)}$ and$DW_{ZZ,cos(\frac{\pi}{6})}$), exhibit most significant current suppression.

For the highly conductive single downward-polarized domain [Fig S10a], the spectrum reveals that integration range of Eq.1 only considers the lowest energy (above E_F) transmission channel ($k_\perp \approx 0.33(\frac{2\pi}{a_0}); E \approx 0.22\,eV$). Transmission eigenstates [Fig S10d] calculated at this channel [cyan square in Fig S10a] have much larger amplitudes in the lower layer MoS_2, which therefore carries the majority of electron current. Since the lower layer of a upward-polarized domain has slightly higher conduction band minimum (CBM) than that of downward-polarized domain, drain current across the upward domain is slightly lower.

Remarkably, the k-space resolved transmission profile is largely preserved in the junction containing $DW_{AC,-cos(0)}$ [Fig S10b]. This type of domain walls (tensile strain at its lower layer) introduces small electronic scattering due

to demoted CBM [Fig S8c,f], thus explaining its high conductance. Transmission eigenstates calculated at its low energy channel [cyan squares marked on Fig S10b] have much larger amplitudes on lower channel, similar to that of downward-polarized channel.

In contrast, the k-space transmission profile of the junction containing $DW_{AC,cos(0)}$ [Fig S10c] is drastically different from that of a highly conductive single downward-polarized domain. Specifically, the transmission channel at $k_\perp \approx 0.33(\frac{2\pi}{a_0})$ is energetically suppressed, with its minimum energy elevated to nearly match that of the secondary channel ($k_\perp \approx 0.2(\frac{2\pi}{a_0})$ and $E \approx 0.4\,eV$). Examining the real-space representation of transmission eigenstates at this channel, we observe amplitude of eigenstates at lower layer greatly diminish near the center of the junction, a region with the highest compressive strain and highest CBM [Fig S8a,d], resulting in substantial electron scattering. Large amplitudes of eigenstates is also observed at the upper layer, suggesting substantial electron transmission through it.

8. ELECTRICAL CURRENT ACROSS MULTI-DOMAIN WALL JUNCTION

Fig S11a illustrate the multi-domain walls structure in a FESFET-junction. Drain current across the junction is calculated as function of drain bias (V_d) under gate voltage $V_G = 0\,V$ [Fig S11a], the suppression of current is dependent on the number, position and types of domain walls within the junction. Specifically, Junction 1 and Junction 2 both have a single domain wall ($DW_{AC,cos(0)}$) that positioned near the left and center of the junction, but $I_{Junction\,2}$ is much smaller than $I_{Junction\,1}$. Junction 3 with two insulating $DW_{AC,cos(0)}$ (positioned near left/center of the junction) and a conductive $DW_{AC,-cos(\frac{\pi}{3})}$ (center of junction) has the lowest current.

(a)

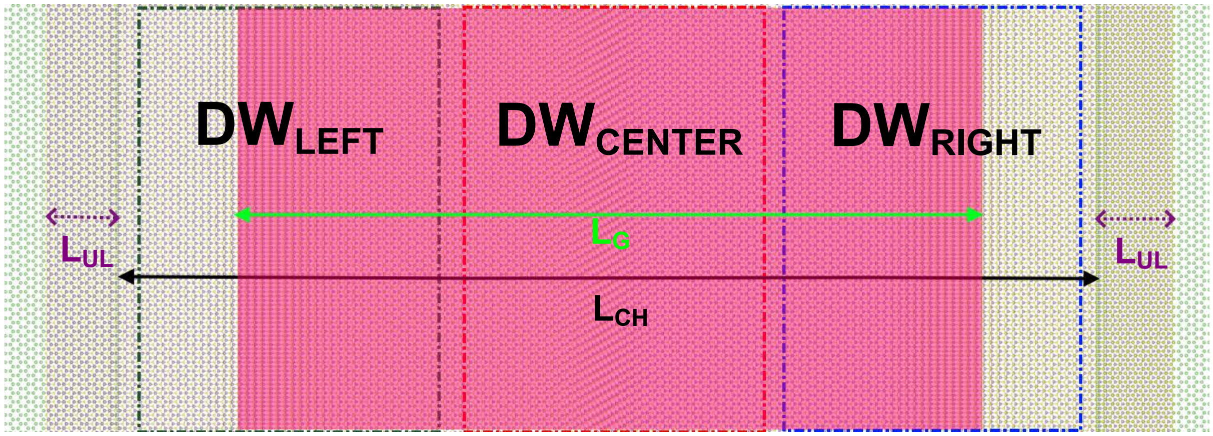

(b)

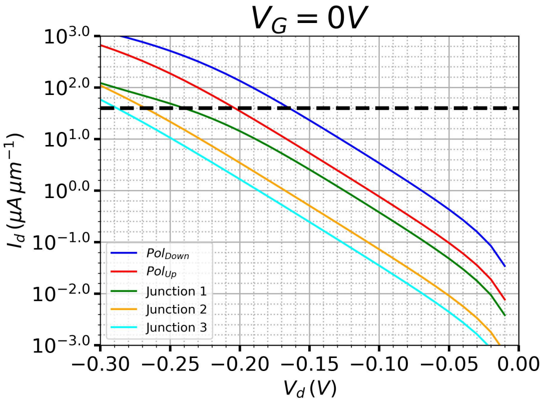

(c)

	DW_{LEFT}	DW_{CENTER}	DW_{RIGHT}
Pol_{Down}	N/A	N/A	N/A
Pol_{Up}	N/A	N/A	N/A
Junction 1	$DW_{ZZ,cos(0)}$	N/A	N/A
Junction 2	N/A	$DW_{ZZ,cos(0)}$	N/A
Junction 3	$DW_{ZZ,cos(0)}$	$DW_{ZZ,-cos(\pi/3)}$	$DW_{ZZ,cos(0)}$

Fig. S11. (a) Top view illustration of the gated bilayer MoS_2 FeSFET with multi-domain walls formed along the armchair axis, within the channel region. Following parameters were used in NEGF calculations: Length of metal gate (L_G) = 30.0 nm, thickness of dielectric (t_ϵ) = 1.0 nm, channel's length (L_{CH}) = 38.0 nm, length of overlapped region between MoS_2 and electrode (L_{UL}) = 2.8 nm. (b) Drain current across the junction as function of drain bias (V_d) under gate voltage $V_G = 0\,V$, referenced value of 40 $\mu A/\mu m$ is plotted as black dashed line. The domain walls formed within the channel region are listed in (c).

9. ELECTRICAL CURRENT ACROSS DOMAIN WALL FORMED BETWEEN AB- AND AA-STACKED $\mathbf{MoS_2}$

Similar to domain walls formed between AB-stacked (upward-polarized) and BA-stacked (downward-polarized) bilayer MoS_2, domain wall between AB-stacked and AA-stacked bilayer MoS_2 is characterized by its normal axis and the angle (θ) between the normal axis and the interlayer sliding displacement, denoted as $DW_{ZZ(AC),cos\theta[AB\,to\,AA]}$. $DW_{AC,cos(0)[AB\,to\,AA]}$ [Fig S12a] has maximum compressive (tensile) strain of 3.8% (4.2%) at its lower (upper) layer while maximum compressive and tensile strain within $DW_{ZZ,cos(\frac{\pi}{2})[AB\,to\,AA]}$ is less than 0.2%. We note that both the atomic sliding and strain are more pronounced near the AA-stacked region. The maximum local strain within $DW_{AC,cos(0)[AB\,to\,AA]}$ is also much larger than the local strain within $DW_{AC,cos(0)[AB\,to\,BA]}$ since AA-stacking has much larger stacking energy, leading to localization of deformation. The larger compressive strain within $DW_{AC,cos(0)[AB\,to\,AA]}$ leads to greater suppression of current [Fig S12b], which $\frac{I_{down}[AB\,to\,AA]}{I_{DW_{AC,cos(0)}}}$ can reach maximum value of 300; minimal strain within $DW_{ZZ,cos(\frac{\pi}{2})[AB\,to\,AA]}$ results in drain current similar to current across AB-stacked junction (I_{up}) [Fig S12c].

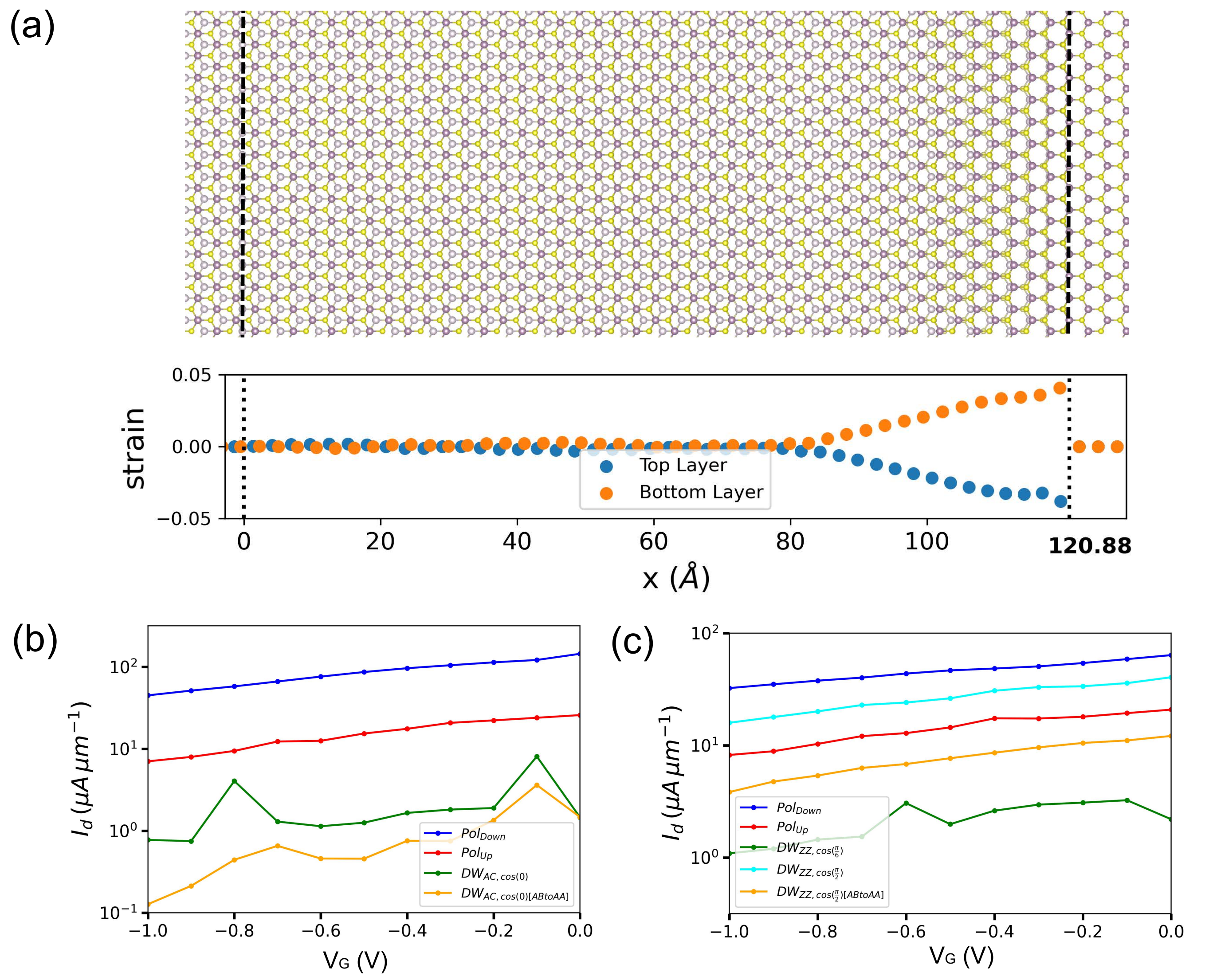

Fig. S12. (a) Top view illustration of the atomic reconstruction within a domain wall formed between AB-stacked (upward-polarized) and AA-stacked bilayer MoS_2, which the domain wall's normal axis is formed along the armchair axis. The domain wall is coined as $DW_{AC,cos(0)[AB\,to\,AA]}$ since sliding displacements of atoms within the domain wall are parallel to the normal axis. The local strain within $DW_{AC,cos(0)[AB\,to\,AA]}$ is illustrated in the lower panel. (b-c) Drain current as function of gate voltage (V_G) across junction along armchair and zigzag axis, respectively.

REFERENCES

[1] S. Smidstrup, T. Markussen, P. Vancraeyveld, J. Wellendorff, J. Schneider, T. Gunst, B. Verstichel, D. Stradi, P. A. Khomyakov, U. G. Vej-Hansen, *et al.*, Quantumatk: an integrated platform of electronic and atomic-scale modelling tools, Journal of Physics: Condensed Matter **32**, 015901 (2019).

[2] J. P. Perdew, K. Burke, and M. Ernzerhof, Generalized gradient approximation made simple, Physical review letters **77**, 3865 (1996).

[3] S. Grimme, Semiempirical gga-type density functional constructed with a long-range dispersion correction, Journal of computational chemistry **27**, 1787 (2006).

[4] M. Büttiker, Y. Imry, R. Landauer, and S. Pinhas, Generalized many-channel conductance formula with application to small rings, Physical Review B **31**, 6207 (1985).

[5] P. S. Bednyakov, B. I. Sturman, T. Sluka, A. K. Tagantsev, and P. V. Yudin, Physics and applications of charged domain walls, npj Computational Materials **4**, 65 (2018).
